# Refractive Index Change of Cellulose Nanocrystal-Based Electroactive Polyurethane by an Electric Field

**DOI:** 10.3389/fbioe.2021.606008

**Published:** 2021-01-28

**Authors:** Jaehwan Kim, Hyun-U Ko, Hyun Chan Kim

**Affiliations:** Department of Mechanical Engineering, Creative Research Center for Nanocelluose Future Composites, Incheon, South Korea

**Keywords:** electroactive polymer actuator, cellulose nanocrystal, tunable lens, refractive index, polyurethane

## Abstract

A tunable optical lens can tune or reconfigure the lens material itself such that it can eliminate the moving part of the lens, which brings broad technological impacts. Many tunable optical lenses have been implemented using electroactive polymers that can change the shape of the lens. However, the refractive index (RI) change of electroactive polymers has not been well investigated. This paper investigated the RI change of CNC-based transparent and electroactive polyurethane (CPPU) in the presence of an actuating electric field. The prepared CPPU was electrically poled to enhance its electro-optical performance, and the poling conditions in terms of frequency and electric field were optimized. The poled CPPU was characterized using a Fourier transform infrared spectroscopy and a refractometer. To investigate the RI change in the presence of an actuating electric field, the poled CPPU was constrained between two electrodes with a fixed distance. The RI linearly increased as the actuating electric field increased. The RI change mechanism and the optimized poling conditions are illustrated. The tunable RI is a promising property for implementing a tunable optical lens.

## Introduction

**Figure 1:**
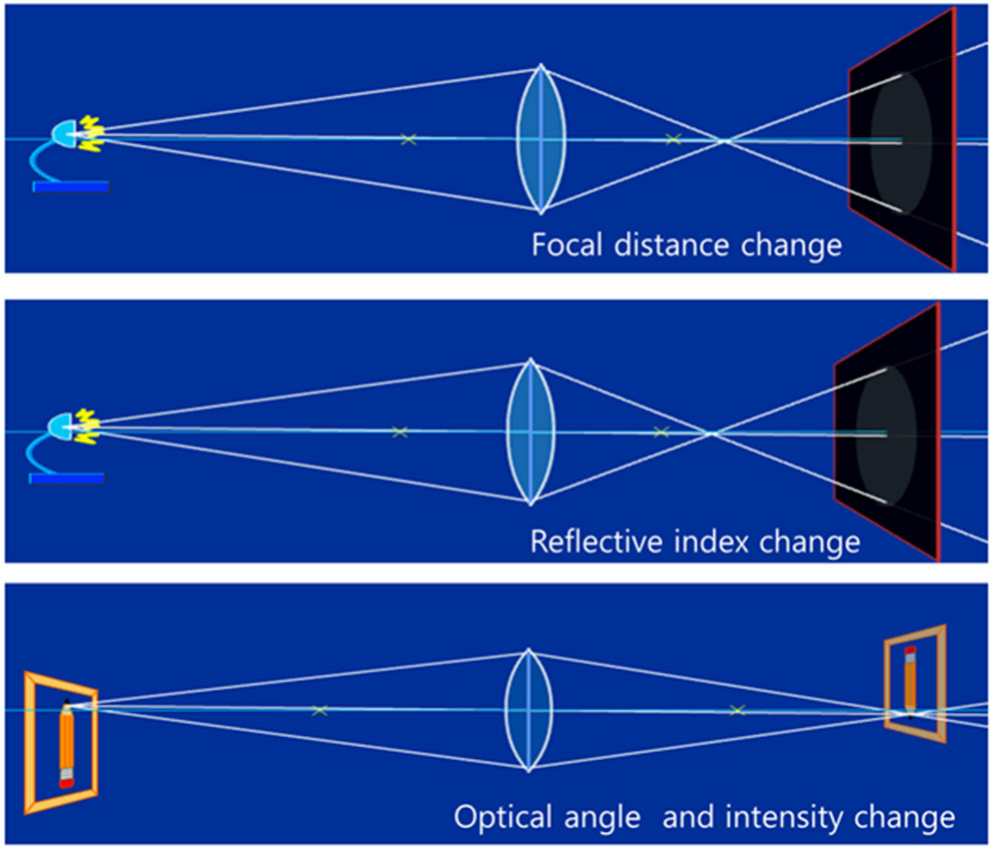
Concepts of a tunable optical lens by focal distance change, refractive index change, and optical angle and intensity change (Choi et al., 2017, republished from IOP).

A tunable optical lens can change the focal distance and refractive index, optical angle, and optical intensity by tuning or reconfiguring the lens material itself (Choi et al., 2017; Ghilardi et al., 2019). Figure 1 shows the concept of a tunable optical lens. A tunable optical lens can eliminate the moving part of a lens, which has a broad technological impact in the industry, military, and consumer products applications such as camera lenses, endoscopes, projectors, membrane optics, telescopes, spectroscopes, and flat-panel displays, etc.

There are two distinct ideas to implement a tunable optical lens by changing the shape of the lens and tuning the refractive index (RI) of the lens material. Shape change of lens materials has been studied in electroactive polymers (EAP) and soft actuator materials (Kim J. et al., 2019). The electrical response, lightweight, and high energy density of EAP make them suitable for biomimetic actuators, soft robots, and optical lenses (Mirfakhrai et al., 2007). There are many available EAPs for tunable optical lens applications such as thermo-responsive polymers (Kim J. et al., 2019), hydrogels (Jayaramudu et al., 2016, 2017), and dielectric elastomer (DE). DE, a kind of EAP, attracts much interest due to its impressive electroactive strain, mechanical robustness, and inexpensive and affordable high energy density, although large operation voltage over is its critical disadvantage (O'Halloran et al., 2008; Zurlo and Destrade, 2017). However, in realizing the tunable optical lens, lens material's actuation performance against repetitive deformation and miniaturization should be carefully considered. Another consideration is the lens material should maintain its transparency from repeated light exposure and actuation. The dielectric property improvement of DE is critical to improving its actuation performance (Romasanta et al., 2015). The dielectric property of DE can be enhanced by blending high dielectric fillers or conductive fillers with dielectric elastomers with different mechanical and dielectric properties (Park et al., 2007; Gallone et al., 2010). However, the choice of dielectric fillers for DE in tunable optical lens research is limited because they should be transparent.

Cellulose nanocrystal (CNC) has been used as fillers of DE actuators and optoelectronics (Giese and Spengler, 2019; Wang et al., 2019). A new strategy was reported to achieve a transparent and reconfigurable actuator by using a DE actuator. Cellulose is a well known and abundant biopolymer globally, which consists of a crystal region and amorphous region. The crystal region, so-called cellulose nanocrystal (CNC), has remarkable advantages such as high mechanical specific strength, biocompatibility, nanoscale dimension, and sustainability (Habibi et al., 2010; Moon et al., 2011; Kim et al., 2015; Vanderfleet and Cranston, 2020; Wang et al., 2020). Typically CNC has lengths of 100–200 nm and cross-sections of 5–20 nm. CNC has been actively studied for many applications, including structural nanocomposites, optical films, and bio-composites. One fascinating property of CNC is its electrical and magnetic polarity (Pullawan et al., 2012). Due to its polarity, CNC can be the right candidate for improving its dielectric property of DE by blending it with polymeric materials without sacrificing polymers' optical transparency. The significance of biodegradable and transparent behaviors of CNC-polymer nanocomposites has been investigated in electronics, optics, and biomedical engineering (Siqueira et al., 2010). Recently, CNC-based transparent and electroactive polyurethane (CPPU) was reported, which is applicable for an actively tunable optical lens (Sadasivuni et al., 2016; Ko et al., 2017). CNC was used for high dielectric filler to improve the electromechanical behavior of CPPU. To achieve good transparency and a homogeneous distribution of CNC in polyurethane, CNC- Poly[di(ethylene glycol) adipate] (PDEGA) was used to play as a polyol. The fabricated CPPU exhibited high transparency (>90%) and 10% of electromechanical strain under 3 V/μm electric field. In this observation, the actuation mechanism was associated with the enhanced electrostatic force of the CPPU by the CNC dielectric filler.

However, a question remained on this actively tunable material. Can RI of CPPU actively change in the presence of an electric field? Recently some researchers reported that RI could be actively tuned by incorporating CNC or any filler materials in the presence of external stimuli. Self-assembled CNC three-layer films with the helicoidal and nematic-like organization of the CNCs were reported (De La Cruz et al., 2018). They exhibited high reflectivity tunable within the visible and near-infrared regions of the optical spectrum. A class of composite polymer films showed the refractive index change by simple mechanical forces (Sandrock et al., 2004). The films were comprised of 1,024 alternating layers of an elastomer and a glassy polymer. The elastomer component's layer thickness and thus, the effective RI of the composite can be varied by compressing the composite. The variable RI mechanism includes both changes in elastomer layer thickness and pressure dependence of the elastomer RI. A set of commercial (meth)acrylic resins was photopolymerized under identical irradiation conditions, and the evolution of their RI was reported that it linearly increased with conversion as long as the material was not in the glassy state (Aloui et al., 2018). This increase was related to a rise in the material density arising during polymerization.

This paper aims at investigating the RI change of CPPU in the presence of an electric field. The CPPU was prepared with different electrical poling conditions of field strength and frequency. Electrical poling is essential to align CNC, which improves the dielectric property of CPPU. Interaction of CNC in the poled CPPU was studied using a Fourier transform infrared spectroscopy (FTIR), and dielectric properties of CPPU were characterized using an LCR meter. RI of the poled CPPU was investigated using a refractometer and a laser displacement sensor. The electro-optical behavior of CPPU was investigated with three different actuation configurations.

## Materials and Methods

### Materials

Micro cellulose, Avicel, was purchased from Sigma-Aldrich, St. Louis, Missouri, USA, and sulfuric acid (H_2_SO_4_) was purchased from JUNSEI chemical in Tokyo, Japan. PDEGA for the soft segment of CPPU and hexamethylene diisocyanate (HMDI) for the hard segment of CPPU were obtained from Sigma-Aldrich. Dibutyltin dilaurate (DBDL) for catalysis of urethane reaction was also purchased from Sigma-Aldrich.

### Fabrication of CPPU

**Figure 2:**
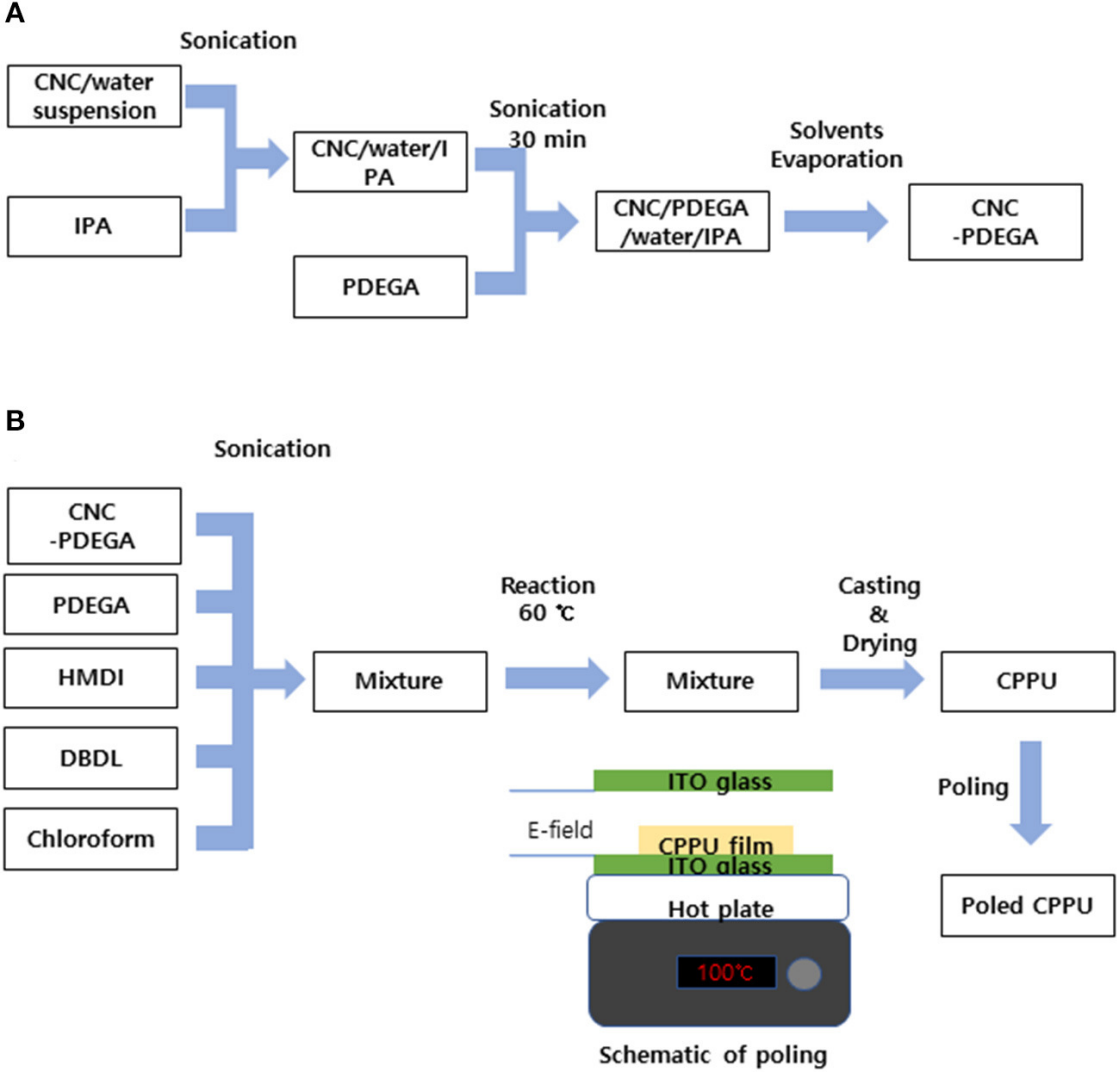
Preparation process of CPPU: **(A)** CNC-PDEGA and **(B)** CPPU preparation and poling.

Figure 2 shows the fabrication process of CPPU. CNC was isolated from micro cellulose using the previously reported acidic hydrolysis process (Kim et al., 2015). In 50 mL of 60 wt% H_2_SO_4_, Avicel was added and heated at 50°C, followed by stirring for 30 min until it turned to light brown suspension. The suspension was diluted with deionized (DI)-water and centrifuged until pH was changed to neutral. The CNC concentration was adjusted to 1 wt%.

The homogeneous distribution of CNC and PDEGA blend (CNC-PDEGA) was prepared before the urethane reaction. Figure 2A shows the process. The preparation process is following (Ko et al., 2017). At first, isopropyl alcohol (IPA) was mixed with CNC suspension at 10:1 ratio using an ultrasonic cleaner (FS30H, Fisher Scientific, Pittsburgh, Pennsylvania, USA) for a few seconds. Then the mixture was added into PDEGA and ran the ultrasonic cleaner for 30 min. The CNC concentration in CNC-PDEGA was varied by 0.001, 0.01, 0.05, and 0.05 wt%. The CNC/IPA/PDEGA solution was heated to removed water and IPA using an oven at 60°C for 48 h. The prepared CNC–PDEGA plays as a polyol in polyurethane. Figure 2B shows the CPPU preparation process. 1 g CNC-PDEGA and 1.25 g PDEGA were dissolved in 5 ml chloroform, and 0.125 g HMDI as an isocyanate salt and 0.1 g DBDL as catalysis for urethane were mixed in the solution. After mixing all chemicals, the solution was encapsulated and kept in the oven at 60°C to form a nucleophilic addition reaction for 5 h. After the reaction, the solution was cooled at room temperature to prevent bubbles generated by evaporating chloroform. The reacted solution was poured on a PDMS sheet and kept in the oven to evaporate the remained chloroform for 1 day. Finally, a solidified CPPU was obtained.

Electrical poling was conducted to enhance the electro-optical performance of CPPU. The prepared CPPU was loaded between two ITO glass electrodes with 5 mm gap. For effective poling, the prepared CPPU was heated at 100°C using a hotplate. The electrical poling signal was generated from a function generator (33220A, Agilent, Santa Clara, California, USA) and amplified using a high voltage amplifier (20/20, Trek, Lockport, New York, USA). Electrical poling field strength and frequency were changed to find an optimum poling condition. At last, a poled CPPU was prepared.

### Characterizations

**Figure 3:**
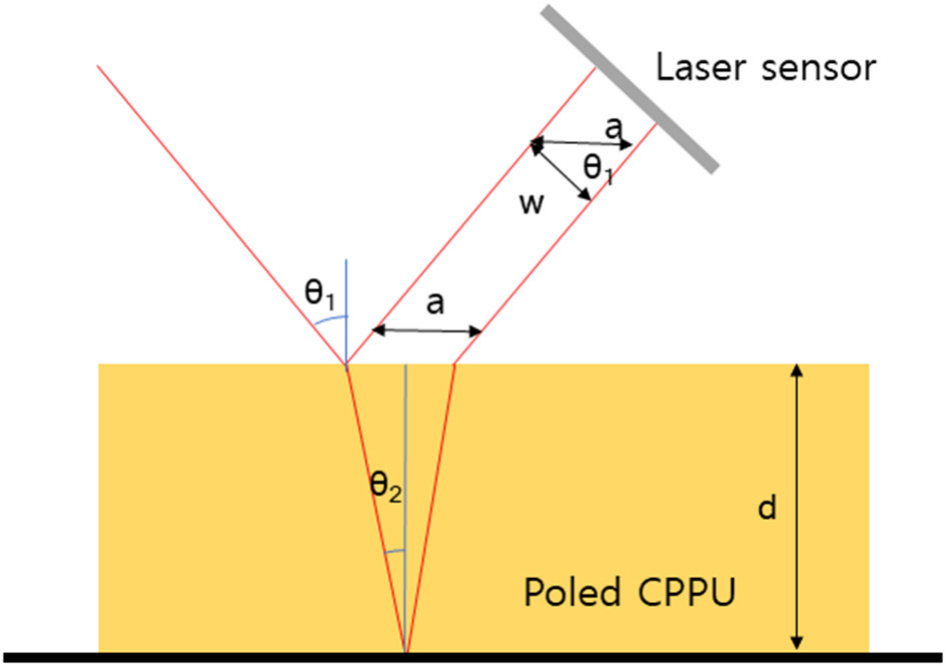
Schematic of laser beam refractions at the surface and bottom of CPPU.

Interaction CPPU in the prepared CPPU was studied using an FTRI (VERTEX 80V, Bruker, Billerica, MA, USA). Dielectric properties were investigated using an LCR meter (Agilent 4284a) with a dielectric measurement fixture (16451b, Agilent, Santa Clara, California, USA). RI of the poled CPPU with various CNC concentration was investigated using a refractometer (DR-M2/1550, ATAGO, Tokyo, Japan). However, the reflectometer does not allow the measurement in the presence of an electric field. Thus, a laser displacement sensor (LK-G15, Keyence, Osaka, Japan) was used to measure the RI change under the electric field. Figure 3 shows the schematic of the displacement sensor's laser light paths in CPPU how to calculate the RI. According to Snell's law, incidence angle θ_1_ and refraction angle θ_2_ have the following relation:

(1) $$\begin{array}{l} \frac{n_{c}}{n_{air}}\approx n_{c}=\frac{sin\theta_{1}}{sin\theta_{2}} \end{array}$$

where n_c_ is the RI of CPPU, and θ_1_ is 37.5°. The incidence angle θ_1_ and refraction angle θ_2_ also have the following relation with *d*, the CPPU thickness, and *w*, measured laser beam distance from the laser displacement sensor between the reflected beams at the bottom surface of CPPU.

(2) $$\begin{array}{l} 2d•tan\theta_{2}=\frac{w}{\cos37\times 5^{◦}} \end{array}$$

(3) $$\begin{array}{l} \theta_{2}={\text{tan}}^{-1}\left(\frac{w}{2d•cos37\times 5^{◦}}\right) \end{array}$$

From Equations (1)–(3), the RI of CPPU can be calculated as

(4) $$\begin{array}{l} n_{c}=\frac{\text{sin}37.5^{◦}}{\text{sin}\left({\text{tan}}^{-1}\left(\frac{w}{2d•cos37\times 5^{◦}}\right)\right)} \end{array}$$

**Figure 4:**
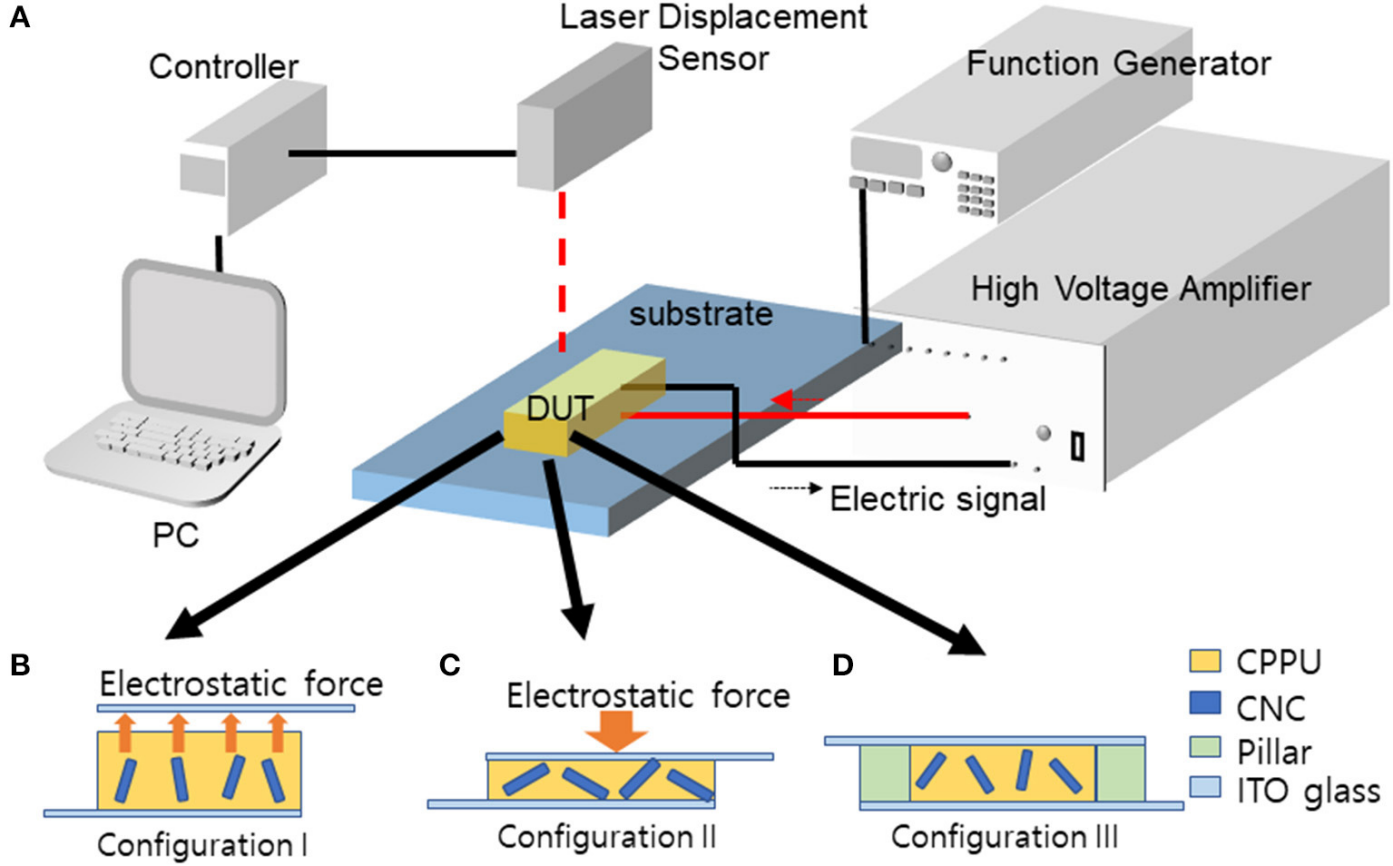
**(A)** Experimental setup for electro-optic behavior and actuation test setup: **(B)** Configuration I, **(C)** Configuration II, **(D)** Configuration III.

The electro-optical behavior of the prepared CPPU was investigated using the experimental setup shown in Figure 4A. An electric signal was generated from the function generator and amplified using the high voltage amplifier. The electric field was applied to CPPU between two ITO glasses. Three different configurations of the ITO glass setup were used, as shown in Figures 4B–D. In the first setup shown in Figure 4B (configuration I), top ITO glass was located over CPPU with 5 mm gap to prevent contact between the CPPU and the top electrode. In the second setup shown in Figure 4C (configuration II), two ITO electrodes contacted the CPPU but no pillars, which allows the free deformation of CPPU in the presence of an electric field. The third setup is shown in Figure 4D (configuration III). Two ITO glasses contacted the CPPU with two pillars, and the distance between the two ITO glasses was fixed. The poled CPPU was cut into 5 × 5 mm^2^. Table 1 shows the test conditions for the electro-optical test. The electrical poling field was changed in 0–200 v/mm with 1–1,000 Hz. The measured RI values were verified by comparing them with the values found using a Mach Zehnder interferometer.

**Table 1:** Actuation and poling conditions for CPPU sample.

**Sample**	**Actuation setup**	**Poling condition**	
		**Frequency (Hz)**	**Electric field (V/mm)**
A	Configuration I	1	20
B	Configuration II	1	20
C	Configuration III	1	20
D	Configuration III	10	20
E	Configuration III	100	20
F	Configuration III	1,000	20
G	Configuration III	1	0
H	Configuration III	1	40
I	Configuration III	1	60
J	Configuration III	1	80
K	Configuration III	1	100
L	Configuration III	1	120
M	Configuration III	1	160
N	Configuration III	1	200

## Results and Discussion

### Characteristics of CPPU With CNC Concentration

**Figure 5:**
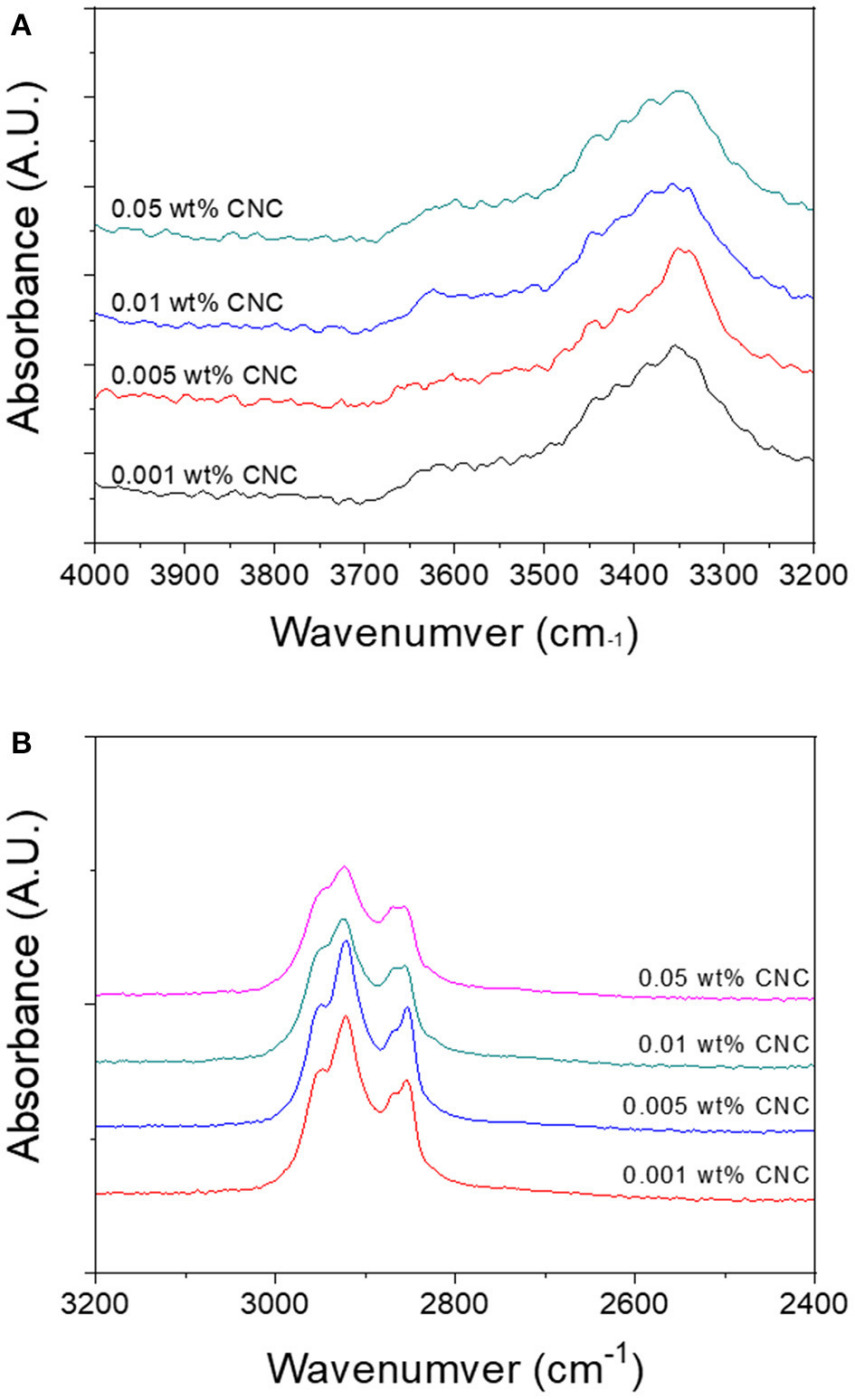
FTIR spectra of poled CPPU with different CNC concentrations at two wavenumber regions: **(A)** 4,000–3,200 cm^−1^ and **(B)** 3,200–2,400 cm^−1^.

FTIR was carried out to investigate the chemical structure of poled CPPU with various CNC concentrations. Figure 5A shows FTIR spectra between 4,000 and 3,200 cm^−1^, and Supplementary Figure 1 represents decomposed FTIR spectra between 3,600 and 3,200 cm^−1^. Different N-H peaks appeared at 3,321, 3,351, and 3,425 cm^−1^, which correspond to ordered hydrogen-bonded N-H, disordered hydrogen-bonded N-H, and free N-H from urethane linkage, respectively (Pullawan et al., 2012). Peaks at 3,390 and 3,425 cm^−1^ correspond to hydrogen-bonded O-H peaks from CNC (Siqueira et al., 2010). A peak at 3,624 cm^−1^ responds to free O-H. As increasing the CNC concentration, the hydrogen-bonded O-H peak increased. Interestingly, disordered hydrogen-bonded N-H peak drastically improved, and ordered N-H peak and free N-H decreased as increasing the CNC concentration. The result might be due to wealthy OH groups in CNC act as hydrogen bonding sites to N-H groups, which are infiltrated between ordered urethane chains and played as an impurity for polyurethane crystal. Moreover, disordered hydrogen-bonded N-H peak shifted to higher wavenumber, 3,349 cm^−1^, which might be due to reduced N-H bonding associated with the lower hydrogen bonding strength of N-HO than N-HN. The peak at 2,920 cm^−1^ shown in Figure 5B corresponds to the C-H stretching peak of alkane structure in polyurethane and CNC. The peak at 2,805 cm^−1^ is the amine peak of polyurethane. As the CNC concentration increasing, 2,948 and 2,867 cm^−1^ peaks corresponding to C-H groups from CNC increased. The FTIR results showed that CNC was well interacted with the amine group of polyurethane by hydrogen bonding, and urethane bonding was well-formed.

**Figure 6:**
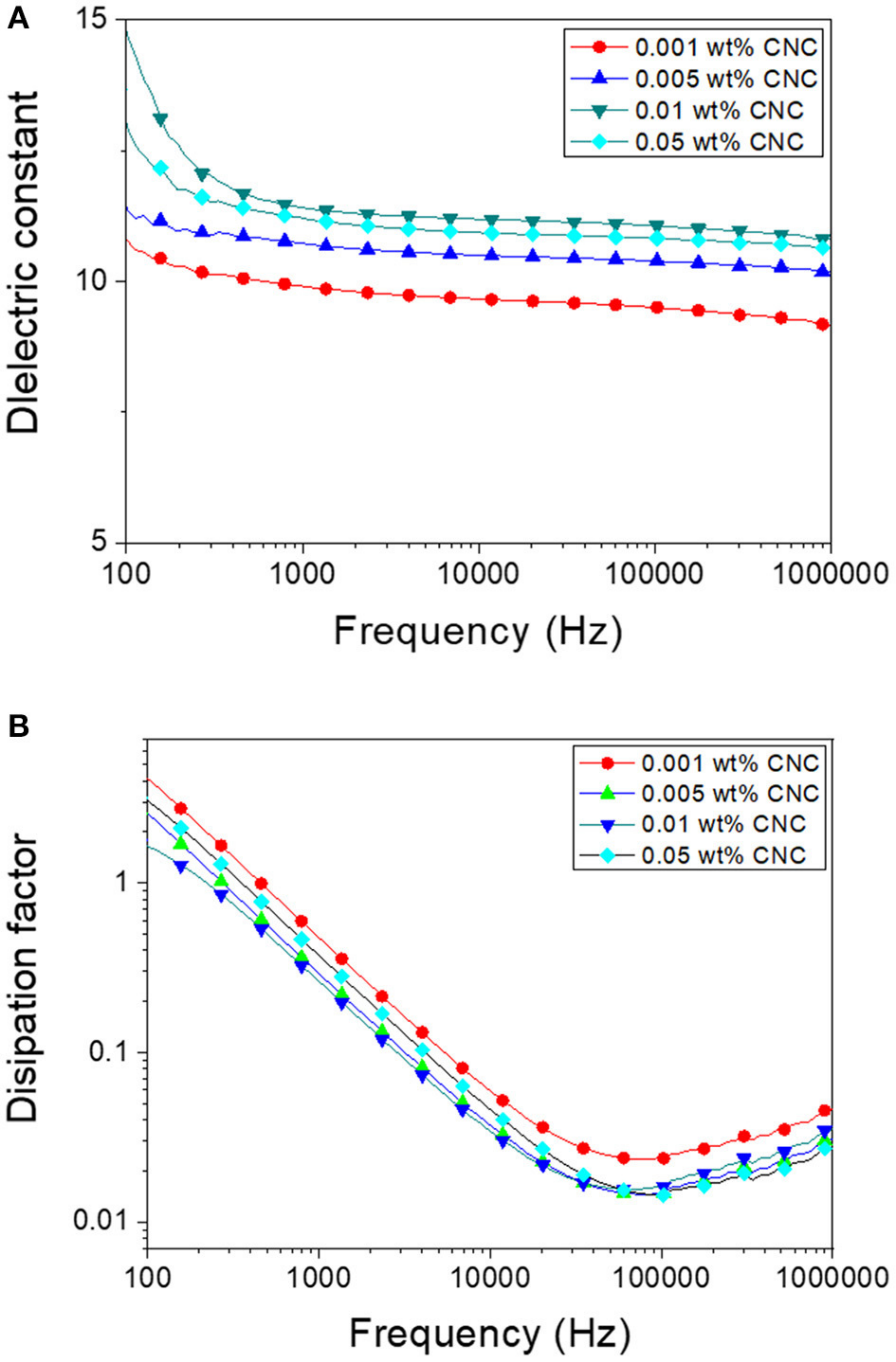
Dielectric properties of poled CPPU with various CNC concentrations: **(A)** Dielectric constant and **(B)** Dissipation factor.

Figure 6 shows the dielectric properties of the poled CPPU with various CNC concentrations. Four polarizations typically govern dielectric properties as surficial, dipole, atomic, and electronic ones. Under GHz range, surficial and dipole polarizations are more dominant than others. The dielectric constant increased as increasing the CNC concentration up to 0.01 wt% and decreased after that (see Figure 6A). By the electrical poling, the dielectric constant increased due to the improved polarization. The dielectric constant at 50 kHz was over 70% of the dielectric constant at 100 Hz. It indicates that the dipole polarization effect in the poled CPPU was more dominant than the surface polarization even at low frequency. Over 0.01 wt%, the rotation of polarized CNC might be limited due to the increased hydrogen bonds between CNC and polyurethane, as shown in the FTIR result. Figure 6B shows dielectric loss curves. As for increasing the CNC concentration, the dielectric loss decreased to 50 kHz, which increased after that. The dielectric loss curves can observe the threshold between surficial and dipole polarization. The dielectric loss result indicates that the surficial polarization effect decreased until 50 kHz. In other words, the dipole polarity was dominant over the point.

Supplementary Figure 2 shows the UV visible curves of the non-poled CPPU and the poled CPPU (Sample C). They delivered over 85% transparency.

The RI of the poled CPPU was investigated before testing the electro-optic behavior. The poling condition was 1 Hz AC with an electric field of 20 V/mm peak to peak, and the CNC concentration was 0.01 wt%. The RI value calculated using Equation (4) was 1.505; meanwhile, the refractometer measurement result was 1.489. It indicates that the RI computed using Equation (4) deviated 1% from the refractometer measurement, an acceptable error range. Supplementary Figure 3 shows the RI of the poled CPPU measured using the refractometer. As increasing the CNC concentration in CNC-PDEGA from 0.001 to 0.05 wt%, the RI rose from 1.48 to 1.51. The RI change might be due to the well-known dielectric filler effect (Aloui et al., 2018). The CNC might be aligned along the electrical poling field.

### Electro-Optical Behavior

**Figure 7:**
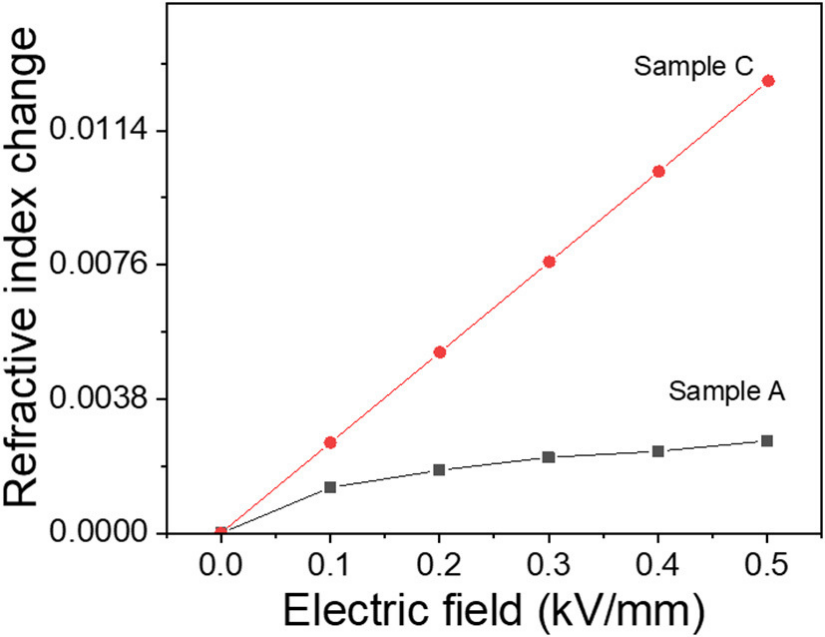
Refractive index changes of configuration I and III with the actuating electric field.

RI change of the poled CPPU with 0.01 wt% CNC concentration was measured using the laser sensor for three configurations shown in Figure 4 in the presence of an actuating electric field. Figure 7 shows the measured RI changes in Configuration I and III. The non-contact electrode case (Configuration I), Sample A, did not show significant RI change as increasing the actuating electric field. It is due to the electrostatic force mitigation between the top electrode and the surface of the CPPU. Supplementary Figure 4 shows the RI result of Configuration II (contact electrode without pillar case), Sample B. As increasing the actuating electric field, the RI slightly decreased, which might be due to the compression of the poled CPPU by the electrostatic force. Initially, CNCs in the poled CPPU aligned along the poling electric field. When the poled CPPU is compressed, CNC could be rotated toward the perpendicular direction to the electric field, which results in the refractive index decrease (Kubo et al., 2007; Zhai et al., 2020). When the poled CPPU is elongated, CNC can be rotated back to the aligned initially and aligned along the electric field. Thus, the sample C (contact electrode with pillar case, Configuration III) was investigated to remove the electrostatic force (compression displacement) effect. In this test, displacement was not changed because of two fixed pillars. As Figure 7 shows, the RI remarkably increased as increasing the actuating electric field. It is the opposite phenomenon with Configuration II (Sample B) due to the CNC alignment along the actuating electric field direction (Kim H. C. et al., 2019).

**Figure 8:**
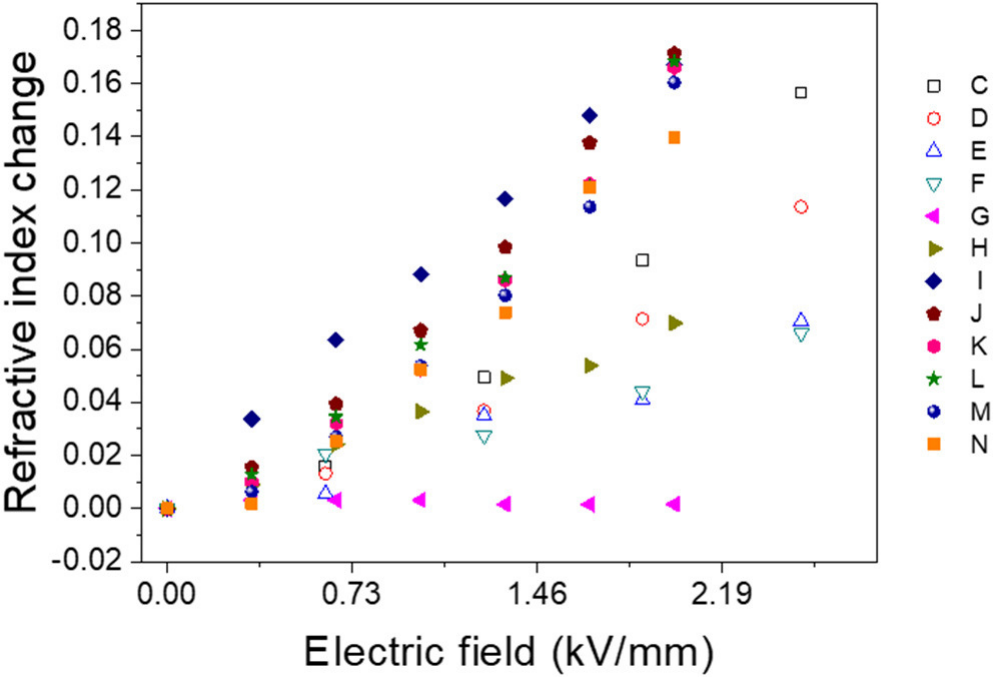
Refractive index change of poled CPPU samples with various poling conditions.

To optimized electro-optical properties, the effect of poling frequency and the poling electric field was investigated with the given conditions in Table 1. Figure 8 shows the RI change results. When the poling frequency increased, the RI change was reduced, and over 100 Hz, the RI change was saturated. The frequency dependence might be associated with the dielectrophoretic effect (Kadimi et al., 2014). The RI increased as increasing the poling electric field up to 60 V/mm and saturated after that. RI change saturation might be due to the optimized CNC alignment (Kubo et al., 2007).

The RI change was demonstrated using a Mach Zehnder interferometer, which compares the laser signal that passes through the poled CPPU with the original signal. Supplementary Video 1 shows interference between two signals, demonstrating a refractive index change of the poled CPPU (Sample I).

The CNC-PDEGA solution can be synthesized for large volume production, and the CPPU can be fabricated by making a urethane bond between CNC-PDEGA and HMDI. After reacting them, the reacted CPPU can be molded for mass production of lens devices.

## Conclusions

This paper investigated the refractive index change of CNC-based transparent and electroactive polyurethane (CPPU) in the presence of an actuating electric field. The prepared CPPU was electrically poled to enhance the electro-optical performance of CPPU. FTIR spectra showed CNC interacted with polyurethane by hydrogen bonding, and urethane bonding was well-formed. The increased CNC concentration increased the dielectric constant of the poled CPPU. Two electrodes were fixed with pillars that contacted the poled CPPU to eliminate the RI change caused by the deformation of CPPU. The RI linearly increased as the actuating electric field increased. Various electrical poling conditions were investigated to optimize the electro-optical behavior, and it was raised as decreasing the poling frequency. The RI change mechanism was associated with the CNC alignment by dielectrophoresis. The RI change increased as increasing the poling electric field up to 60 V/mm and saturated after that. It was due to CNC alignment was optimized at 60 V/mm poling electric field. The results proved that RI is tunable in the presence of the poling electric field, which is a promising property for implementing a tunable optical lens.

## Data Availability Statement

The raw data supporting the conclusions of this article will be made available by the authors, without undue reservation.

## Author Contributions

JK and H-UK: conceptualization, literature review, figures and graphs, and draft. H-UK: experiment. HK: data acquisition. JK: editing. All authors contributed to the article and approved the submitted version.

## Conflict of Interest

The authors declare that the research was conducted in the absence of any commercial or financial relationships that could be construed as a potential conflict of interest.
