# Supplementary material for: Refractive Index Change of Cellulose Nanocrystal-Based Electroactive Polyurethane by an Electric Field
Source: Front Bioeng Biotechnol. 2021 Jan 28;9:606008. doi: 10.3389/fbioe.2021.606008 (PMC7901916; doi:10.3389/fbioe.2021.606008)
Supplement: Supplementary file 1 [file Table_1.DOCX]

Supplementary Material

Refractive index change of cellulose nanocrystal-based electroactive polyurethane by an electric field

Jaehwan Kim*, Hyun-U Ko, Hyun Chan Kim

Creative Research Center for Nanocellulose Future Composites, Inha University, Incheon 22212, Republic of Korea

*Corresponding author: [jaehwan@inha.ac.kr](mailto:jaehwan@inha.ac.kr), Tel: +82-32-960-7326

**
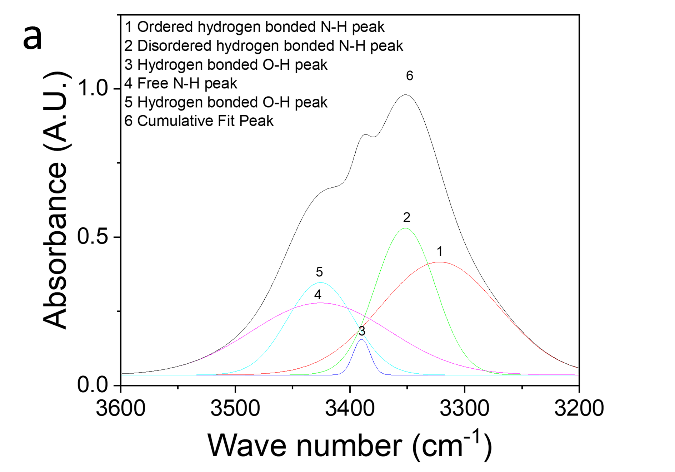

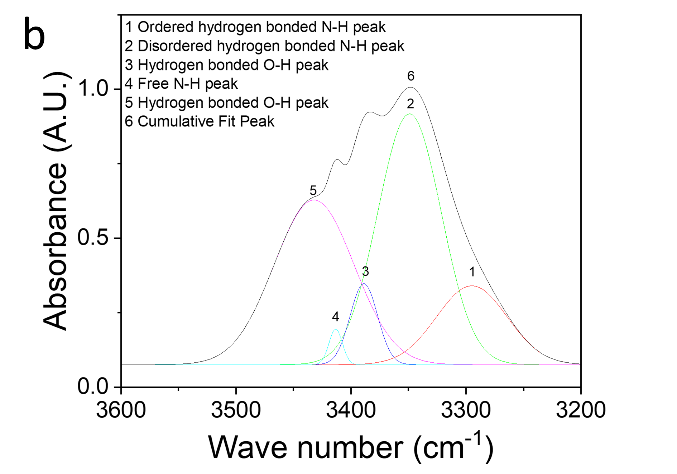
**

**Supplementary Figure 1.** Decomposed FTIR spectra between 3600 and 3200 cm^-1^ for: **a** 0.001 wt% CNC case and **b** 0.05 wt% CNC case.

**
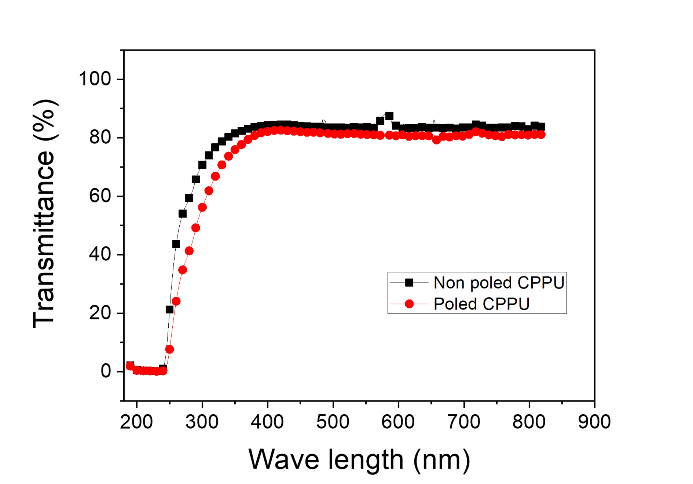
**

**Supplementary Figure 2.** UV-visible spectra of non-poled and poled CPPU.

**
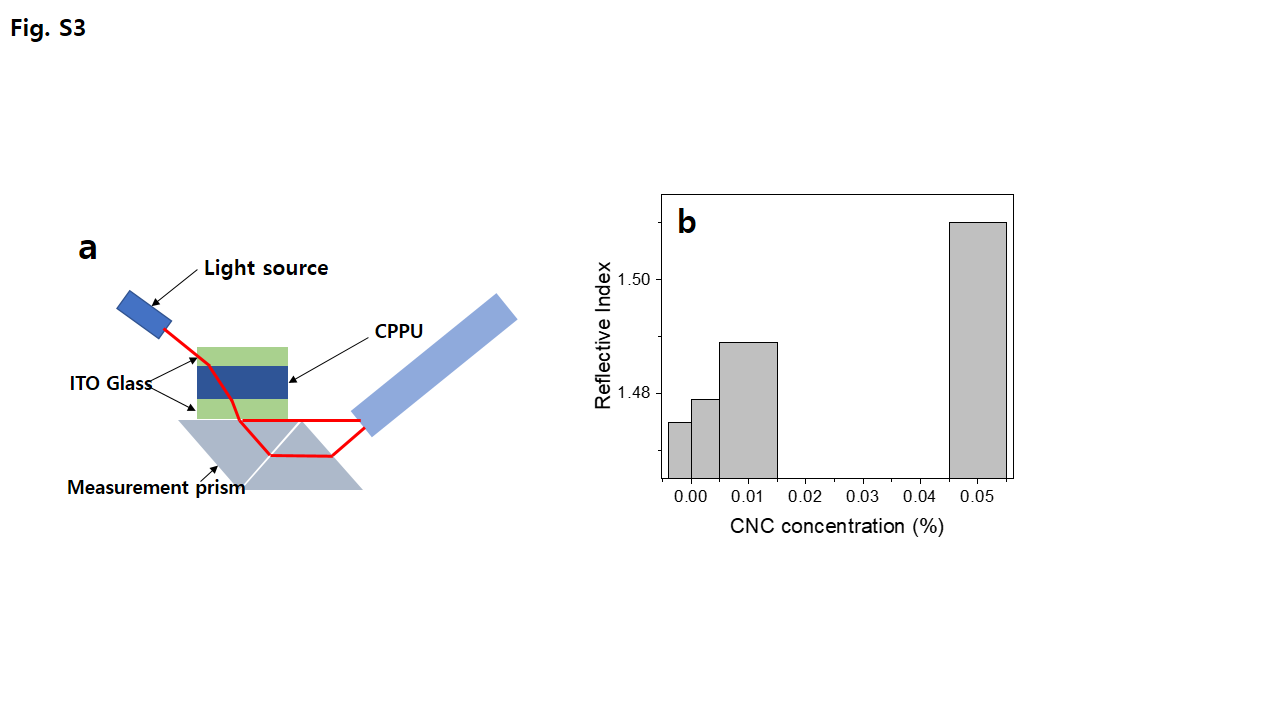
**

**Supplementary Figure 3.** Refractive index change of poled CPPU with CNC concentration: **a** The RI measurement schematic using the refractometer, and **b** the RI change with the CNC concentration.

**
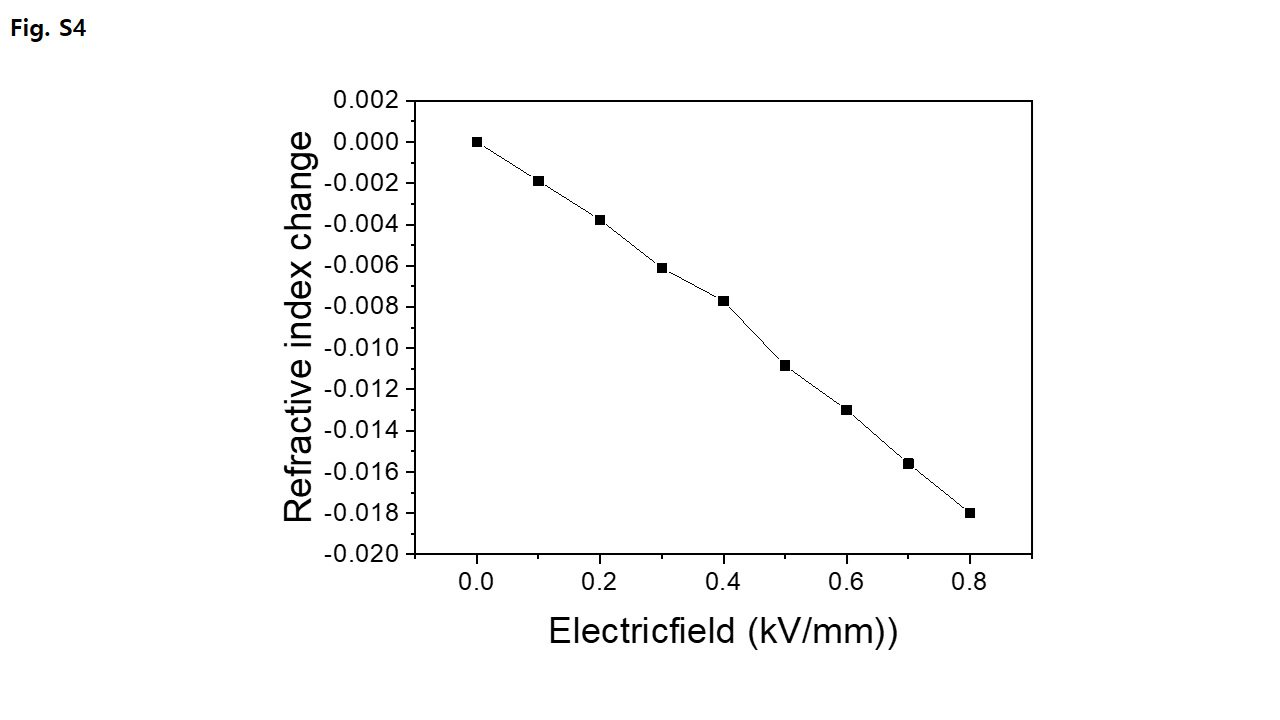
**

**Supplementary Figure 4.** Refractive index change of Configuration II, Sample B.

**
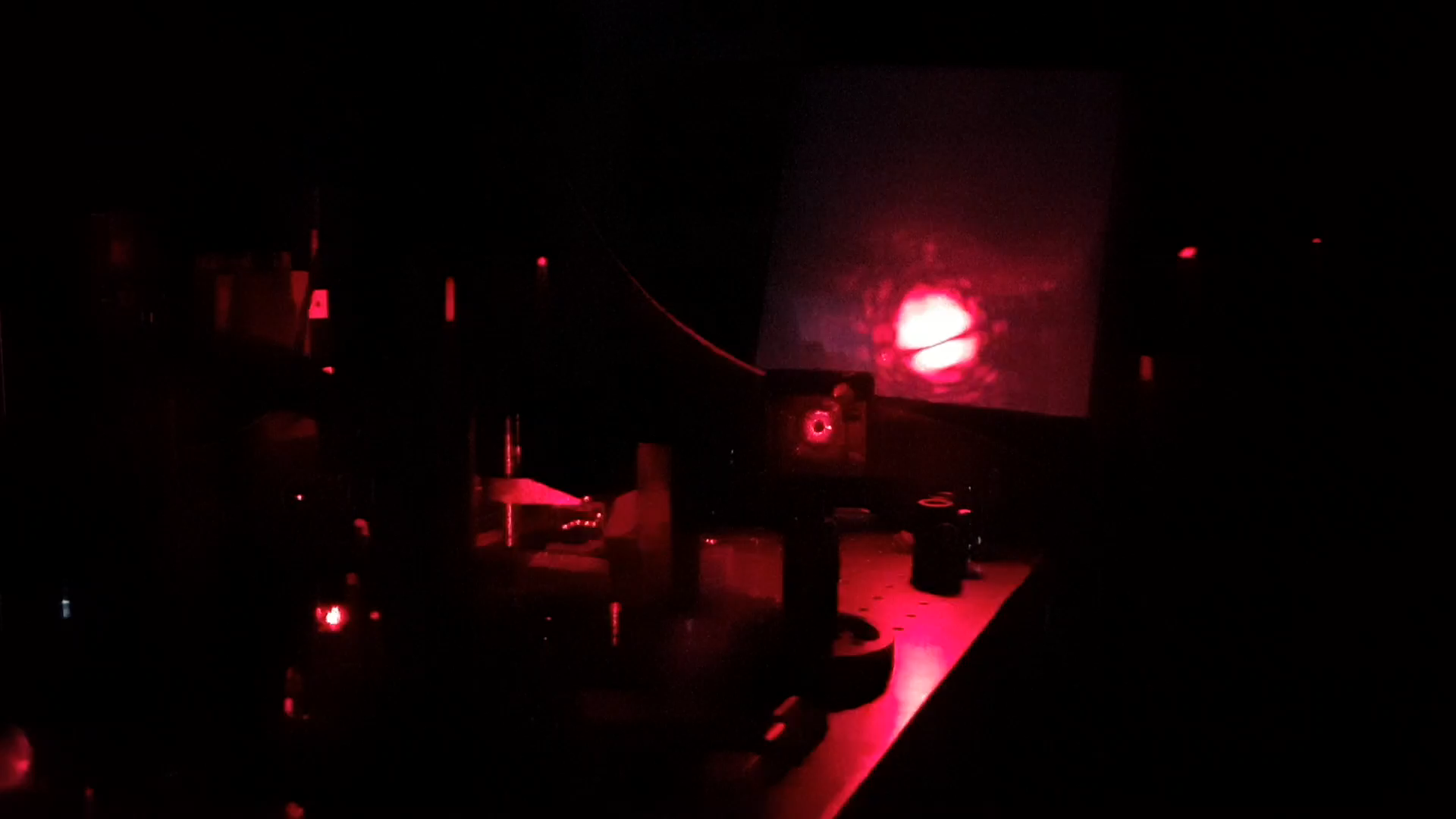
**

**Supplementary Video 1.** Demonstration of refractive index change by Mach Zehnder interferometer. Mach Zehnder interferometer was set up on an anti-vibration table. The screen was used to observe the birefringence of the laser beam (λ = 633 nm) through the poled CPPU (thickness, t =300 μm) and the original laser beam. The poled CPPU was Sample I (poling electric field: 60 kV/mm and 1 Hz). The actuation electric field was 2 kV/mm and 0.5 Hz. The number of fringe changes, P, was 2. From the number and Equation (S2), the RI change was found to be 0.004. ΔOptical Path Length = P λ = Δn〮t; Δn= P λ/t. This result confirmed that the RI could be changed by an electric field without deformation change the poled CPPU. The RI change deviation between the laser sensor measurement result and the Mach Zehnder interferometer one might be due to signal difference in DC and AC.
